# Energy Transfer Studies in Tb^3+^-Yb^3+^ Co-Doped Phosphate Glasses

**DOI:** 10.3390/ma14226782

**Published:** 2021-11-10

**Authors:** Hadil Benrejeb, Kevin Soler-Carracedo, Antonio Diego Lozano-Gorrín, Sana Hraiech, Inocencio Rafael Martin

**Affiliations:** 1 Chemistry Department, Faculty of Sciences of Bizerte, Carthage University, Tunis 7021, Tunisia; 2 Physical Chemistry Laboratory of Mineral Materials and Their Applications, National Center of Research in Materials Science, B.P. 73, Tunis 8027, Tunisia; sanahraiech@gmail.com; 3 Physics Department, University of La Laguna, E-38200 San Cristóbal de La Laguna, Santa Cruz de Tenerife, Spain; ksolerca@ull.edu.es (K.S.-C.); imartin@ull.edu.es (I.R.M.); 4 Chemistry Department, University of La Laguna, E-38200 San Cristóbal de La Laguna, Santa Cruz de Tenerife, Spain; adlozano@ull.edu.es; 5 Institute of Materials and Nanotechnology (IMN), University of La Laguna, E-38200 San Cristóbal de La Laguna, Santa Cruz de Tenerife, Spain

**Keywords:** rare earth, decay curves, up-conversion, down-conversion, optical properties

## Abstract

Detailed optical properties of Tb^3+^-Yb^3+^ co-doped phosphate glasses were performed based on their emission spectra and decay measurements. Under blue excitation of Tb^3+^ at 488 nm, the intensity of Yb^3+^ emissions gradually enhanced upon increasing the Yb^3+^ content until 1 mol% indicated an energy transfer from Tb^3+^ to Yb^3+^. Otherwise, under near infrared excitation of Yb^3+^ at 980 nm, these glasses exhibit intense green luminescence, which led to cooperative sensitization of the ^5^D_4_ level of Tb^3+^ ions. A cooperative energy transfer mechanism was proposed on the basis of the study on the influence of Yb^3+^ concentration on up-conversion emission intensity, as well as the dependence of this up-conversion intensity on near infrared excitation power. Moreover, the temporal evolution of the up-conversion emissions have been studied, which was in positive agreement with a theoretical model of cooperative up-conversion luminescence that showed a temporal emission curve with rise and decay times of the involved levels.

## 1. Introduction

Over the past few decades, great attention has been given to synthesize new luminescent materials activated by lanthanide ions, which allows for efficient energy conversion in different materials. These include down-conversion and up-conversion processes [1,2,3,4] in order to provide a wide range of applications, such as in white light-emitting diodes, displays, medical imaging, infrared lasers, optical telecommunication, solar cells, and fiber amplifiers [5,6,7]. The down-conversion that was predicted by Dexter [8] in the 1950s, converts one ultraviolet visible photon into two near infrared photons [9,10]. Numerous works devoted to down-conversion processes have been reported, such as Ce^3+^-Yb^3+^, Nd^3+^-Yb^3+^ [11], Pr^3+^-Yb^3+^ [12], and Tb^3+^-Yb^3+^ [13]. In these systems the trivalent Yb^3+^ ions act as an energy acceptor in order to emit near infrared photons and achieve spectral conversion due to their electronic configuration. These ions have a simple energy level scheme that consists of a broad absorption of ^2^F_7/2_→^2^F_5/2_ transition in the near infrared range [9,13,14]. Among lanthanide ions, Tb^3+^ is an attractive activator ion, as it can absorb high energy photons and transmit energy to two Yb^3+^ ions. Moreover, the Tb^3+^ ions can be excited by a cooperative energy transfer from two Yb^3+^ ions [15,16], and realizing an up-conversion process that involves two photons from the near infrared range that are then converted to a photon with a higher energy.

The choice of host material is also important for the efficiency of the energy transfer process. The phosphate glass is an attractive host material because of its low melting temperature and excellent chemical stability, as well as its high ultraviolet transmission and allowance of a high concentration of rare earth ions doping; as such, it could be an excellent down- and up- conversion luminescence matrix material. Therefore, the present work focuses on the energy transfer processes between Yb^3+^ and Tb^3+^ analyzing on the photoluminescence and the temporal luminescence curves in order to identify the involved mechanisms.

## 2. Experimental

Phosphate glasses samples were prepared by a conventional melt-quenching method with the following compositions: (37.5–x) P_2_O_5_-40Na_2_O_3_-18ZnO-2Al_2_O_3_-2.5Tb_2_O_3_-xYb_2_O_3_ (in mol%, *x =* 0.1, 0.5, 1, 2 and 3). The stoichiometric chemicals were mixed homogeneously using a mortar and melted in a platinum crucible at 1400 °C for 3 h. The glasses obtained were annealed at 200 °C for 12 h and slowly cooled down to room temperature.

The emission spectra were obtained by exciting the sample with light from a 300 W Xe arc lamp passed through a 0.25 Spex 1681 monochromator (SR-500i-B2-R, Andor Technology, Belfast, UK) with a R928 model for the visible Hamamatsu photomultiplier (Hamamatsu Photonics, Hamamatsu City, Japan) and a R406 model for the near infrared. All spectra were corrected for the instrumental response. Up-conversion spectra were obtained by exciting the samples with a continuous diode laser at 980 nm. The intensity decay measurements were obtained by exciting with a 10 ns pulsed optical parametric oscillator laser (EKSPLA/NT342/3/UVE, EKSPL, Vilnius, Lithuania) and the emission was focused into the entrance of a monochromator, which was coupled to a photomultiplier (a R928 Hamamatsu in the visible range) and a digital oscilloscope (the LeCroy Wave Surfer 424 was used as a detection system, A TEKTRONIX-2430A, Tektronix, Beaverton, OR, USA).

## 3. Theoretical Introduction for the Cooperative Up-Conversion

**Figure 1:**
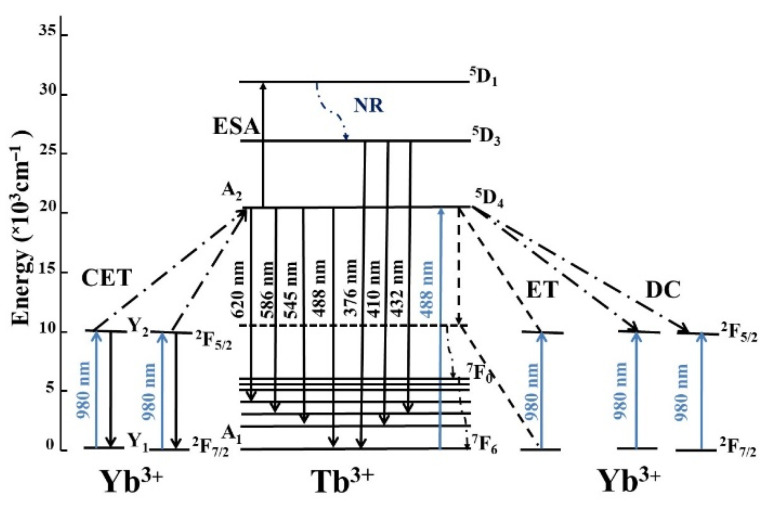
Energy level diagram of Yb^3+^ and Tb^3+^ ions co-doped phosphate glass showing the possible mechanism of energy transfer processes.

At present, there are many studies on Tb^3+^-Yb^3+^ ions co-doped up-conversion materials in which ones the energy can be transferred from a pair of interacting Yb^3+^ ions to an acceptor Tb^3+^ ion. In this up-conversion process, initially two Yb^3+^ ions are excited from the ground ^2^F_7/2_ level to an excited ^2^F_5/2_ state, then this pair of excited Yb^3+^ ions transfers their energy to a Tb^3+^ ion in order to populate the ^5^D_4_ level [17]. This cooperative up-conversion energy transfer process (CET) is showed schematically in Figure 1.

The cooperative energy transfer process from Yb^3+^ to Tb^3+^ can be analyzed in the framework of a rate equation model. In the case of rapid migration among donors, the dynamics of the up-conversion processes produced by cooperative transfer can be described using the following rate equation model [15]:

(1) $$\frac{{\mathrm{dY}}_{2}}{\mathrm{dt}}={\sigma \Phi Y}_{1}-\frac{1}{\tau_{D}}Y_{2}-{\mathrm{WY}}_{2}^{2}A_{1}$$

(2) $$\frac{{\mathrm{dA}}_{2}}{\mathrm{dt}}=-\frac{1}{\tau_{A}}A_{2}+{\mathrm{WY}}_{2}^{2}A_{1}$$

where Y_i_ and A_i_ represent the populations of the ith level for the donors (Yb^3+^) and acceptors (Tb^3+^), respectively, σ is the absorption cross section of excited ions (donor), Φ is the incident pumping flux, W is the cooperative energy transfer rate, and τ_D_ and τ_A_ are the donors and acceptors lifetimes, respectively.

If the ground state depopulation and the transfer term were neglected in Equation (1), then in the steady state condition the population A_2_ can be expressed as:

(3) $$A_{2}=\tau_{A}\tau_{D}^{2}W{\left(\sigma \Phi \right)}^{2}C_{A}C_{D}^{2}$$

where C_D_ and C_A_ correspond to the donor and acceptor concentrations, respectively. According to Equation (3), it is remarkable that the intensity of the up-conversion emission is proportional to the quadratic donor concentration and the pump intensity.

After pulsed excitation at t = 0, the temporal evolution of the up-conversion emission can be described using Equations (1) and (2), and the solution is:

(4) $$A_{2}\left(t\right)=\frac{{\mathrm{WC}}_{A}Y_{2}{\left(0\right)}^{2}}{\frac{1}{\tau_{A}}-\frac{2}{\tau_{D}}}\left[\exp(-2t/\tau_{D})-\exp\left(-t/\tau_{A}\right)\right]$$

where Y_2_(0) is the initial population of excited donor ions. It can be noted that the shape of the transient A_2_(t) (given by Equation (4)) shows a curve with a rise time τ_D_/2 and a decay time τ_A_.

## 4. Results and Discussion

### 4.1. Energy Transfer from Tb^3+^ to Yb^3+^

#### 4.1.1. Luminescence Spectra

**Figure 2:**
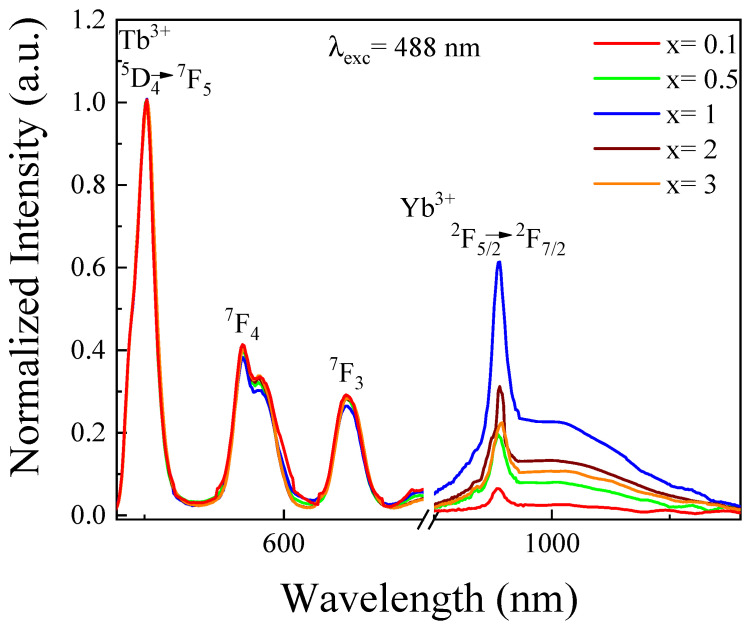
Emission spectra of 2.5Tb^3+^-xYb^3+^ co-doped phosphate glasses normalized to ^5^D_4_→^7^F_5_ peak.

The emission spectra were measured under excitation at 488 nm in 2.5Tb^3+^-xYb^3+^ co-doped phosphate glasses (see Figure 2). It was noticed that the emission spectra showed intense bands in the visible and near infrared ranges at 545, 586, 620, and 980 nm, which were generated by the transitions ^5^D_4_→^7^F_5_, ^7^F_4_, and ^7^F_3_ of Tb^3+^, and ^2^F_5/2_→^2^F_7/2_ of Yb^3+^, respectively. It was noteworthy that the intensity of Yb^3+^ emissions gradually enhanced up on increasing the Yb^3+^ content [18]. This result indicated the presence of energy transfer processes; therefore, a portion of Tb^3+^ ions transferred their energy in order to excite Yb^3+^ ions [13,19]. The Tb^3+^ ions were considered as sensitizer by absorbing visible photons and transferring their energy to Yb^3+^ (which acted as activators). Moreover, with higher Yb^3+^ concentrations that exceeded 1 mol%, it was observed that the near infrared emission intensity reduced significantly. This quenching effect will be analyzed in the following section.

#### 4.1.2. Luminescence Decays

**Figure 3:**
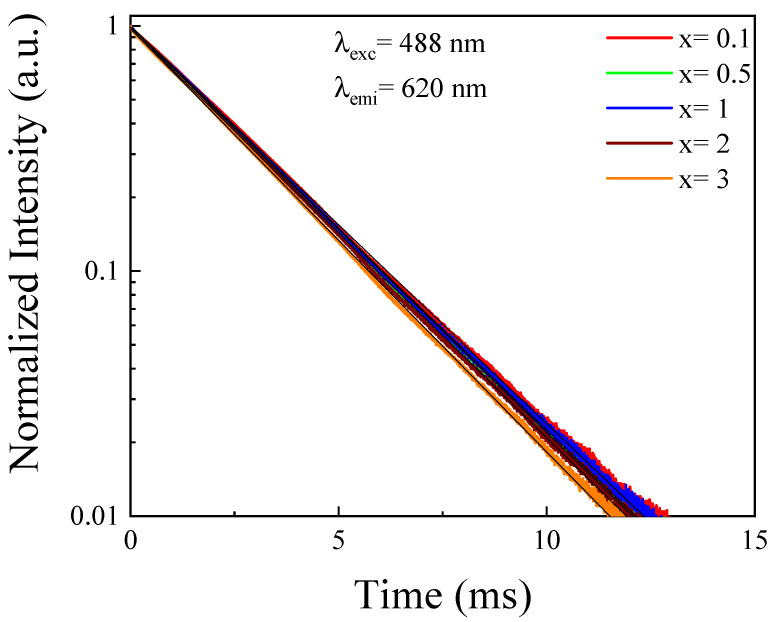
Decay curves of Tb^3+^ in 2.5Tb^3+^-xYb^3+^ co-doped phosphate glasses.

To gain further insight in the energy transfer processes between Tb^3+^ and Yb^3+^, the Tb^3+^ decay curves were obtained exciting at 488 nm and detecting the emission at 620 nm corresponding to ^5^D_4_ levels in phosphate glasses co-doped with different Yb^3+^ concentrations. The results were obtained and shown in Figure 3. It was observed that the lifetime of the ^5^D_4_ level weakly decreased (from 2.7 ms to 2.6 ms) with the increasing of Yb^3+^ concentration doping. Therefore, the Tb^3+^:^5^D_4_ level can be partially depopulated through the energy transfer process due to the increasing of Yb^3+^ acceptor ions [20]. This result could explain the increase of the Yb^3+^ emission obtained in Figure 2.

**Figure 4:**
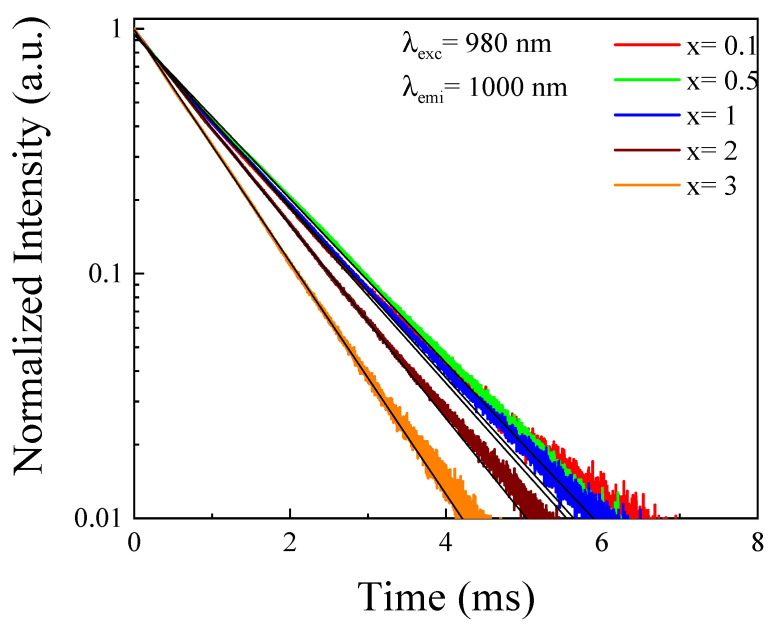
Decay curves of Yb^3+^ in 2.5Tb^3+^-xYb^3+^ co-doped phosphate glasses.

Figure 4 exhibited the decay curves of Yb^3+^:^2^F_5/2_ levels in the 2.5Tb^3+^-xYb^3+^ co-doped phosphate glasses under direct excitation of this level under 980 nm. The decay curves are nonexponential and they can be fitted to the parent model [21] as shown:

(5) $$I\left(t\right)=I\left(0\right)\mathrm{Exp}\left[-t/\tau -Q{\left(t/\tau \right)}^{\frac{3}{S}}-\mathrm{Wt}\right]$$

where I(0) is the intensity at time t = 0, Q is the energy transfer parameter (between donor and acceptor), and W depends on the transfer among donors (migration). When the Yb^3+^ concentration was increased, the decay curves were faster due to an increase of the W parameter, indicating that the migration among Yb^3+^ ions gained in importance. The decay curves for the samples co-doped with 0.1 and 3 mol% were well fitted to the parent model, considering an interaction character dipole–dipole (S = 6) with values of W = 0 and 0.37 ms^−1^, respectively. This energy migration process explained the decrease obtained in the Yb^3+^ emission shown in Figure 2, when the concentration was higher than 1 mol% [20]. However, the main objective in our work was to fit the temporal up-conversion curves, and for this reason the decay curves shown in Figure 4 were fit to exponential. The obtained lifetimes decreased from 1.2 ms to 0.9 ms with the Yb^3+^ concentration; as such, this effect confirmed the previous result of energy migration. An excited Yb^3+^ ion transferred its energy to another non-excited Yb^3+^ ion, and finally, this energy could be transferred to a trap, or Tb^3+^ ions, at lower levels.

**Figure 5:**
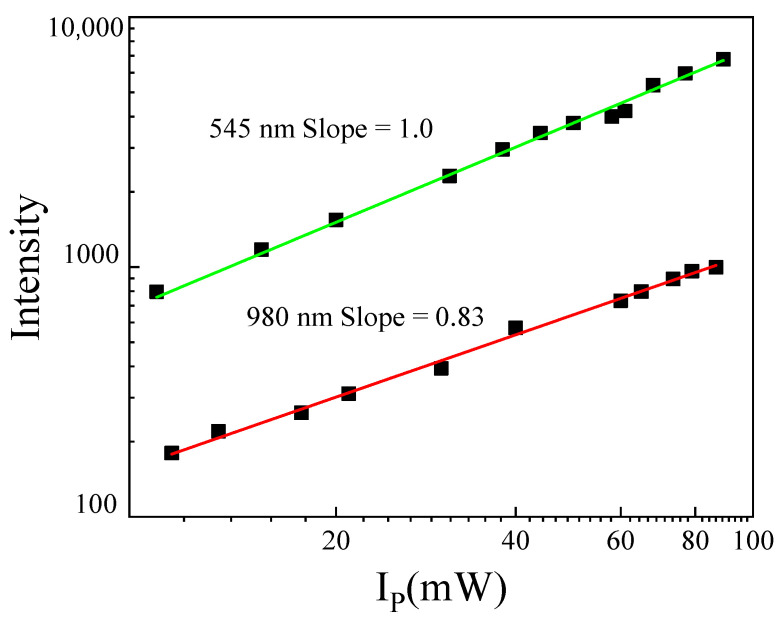
Plot of the Tb^3+^and Yb^3+^ emission intensity as a function of pumping power (Ip) at 488 nm obtained in 2.5Tb^3+^-1Yb^3+^ co-doped phosphate glass.

To get more information about the energy transfer mechanism from Tb^3+^ to Yb^3+^, the excitation power measurements for 2.5 Tb^3+^-1Yb^3+^ co-doped phosphate glass were performed and shown in Figure 5. The slope value for Tb^3+^:^5^D_4_→^7^F_5_ emission is 1.0, exhibiting a linear relationship between the intensity of Tb^3+^ luminescence and excitation power. However, according to the power dependence property for the luminescence of Yb^3+^, the slope value for Yb^3+^:^2^F_5/2_→^2^F_7/2_ was 0.83. Duan et al. developed a Quantum Cutting (QC) model using rate equations in order to explain the sublinear slopes [9]. The slope of Yb^3+^ intensity power dependence was between 0.5 and 1, which indicated the coexistence of two different energy transfer mechanisms in Tb^3+^-Yb^3+^ co-doped phosphate glass, such as one photon process where the Yb^3+^ emission intensity exhibited a linear relation with the excitation power (theory slope of 1.0). This mechanism consists of our system being excited to the ^5^D_4_ level transfer of its energy (ET) to one Yb^3+^ ions by means of a virtual level (Figure 1). From this state, the system relaxed to lower states of Tb^3+^ ions [20]. The second mechanism required a two photons process (theory slope of 0.5), considering that the energy of Tb^3+^ transition (^5^D_4_→^7^F_5_) is approximately twice to Yb^3+^ transition (^2^F_5/2_→^2^F_7/2_). Then the Tb^3+^ ion excited to ^5^D_4_ level could transfer the energy to two Yb^3+^ ions via down-conversion process (DC) emitting two 980 near infrared photons (Figure 1).

### 4.2. Cooperative Energy Transfer from Yb^3+^ to Tb^3+^

#### 4.2.1. Up-Conversion Luminescence Spectra

**Figure 6:**
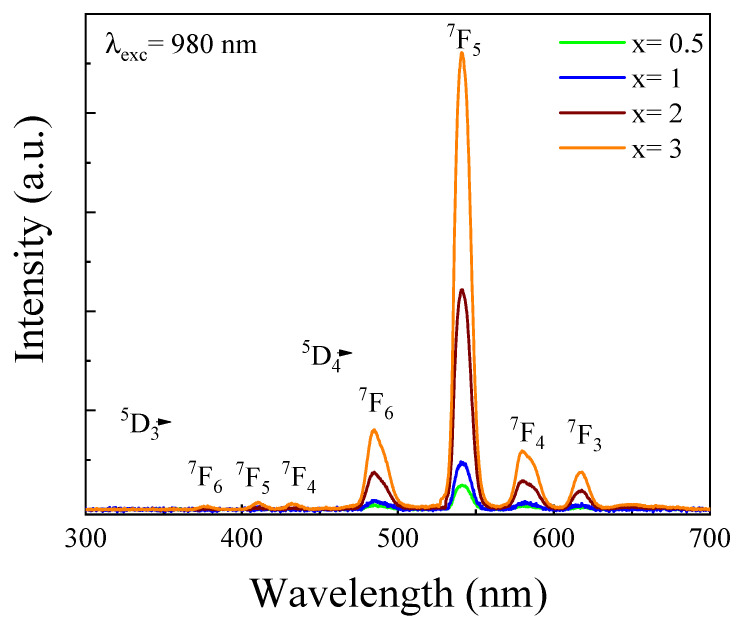
Emission spectra of 2.5Tb^3+^-xYb^3+^ co-doped phosphate glasses.

Figure 6 illustrated the emission spectra of 2.5Tb^3+^-xYb^3+^ co-doped phosphate glasses under excitation at 980 nm. The visible emission showed luminescence bands associated with the f–f electronic transitions of Tb^3+^ ions. It is interesting to note the presence of four peaks at 488, 545, 586, and 620 nm due to radiative relaxation ^5^D_4_ energy level to the ^7^F_6_, ^7^F_5_, ^7^F_4_, and ^7^F_3_, respectively. Surprisingly, with high Yb^3+^ concentration the emission spectra occurred not only from the ^5^D_4_, but also from other levels such as ^5^D_3_. As such, weak bands were detected centered at 376, 410, and 432 nm, resulting from ^5^D_3_→^7^F_6_, ^7^F_5_, and ^7^F_4_ transitions, respectively. It is noteworthy that these transitions were rarely observed under 980 nm excitation. Therefore, this result confirmed the existence of energy transfer from Yb^3+^ to Tb^3+^ via an up-conversion process [22,23].

**Figure 7:**
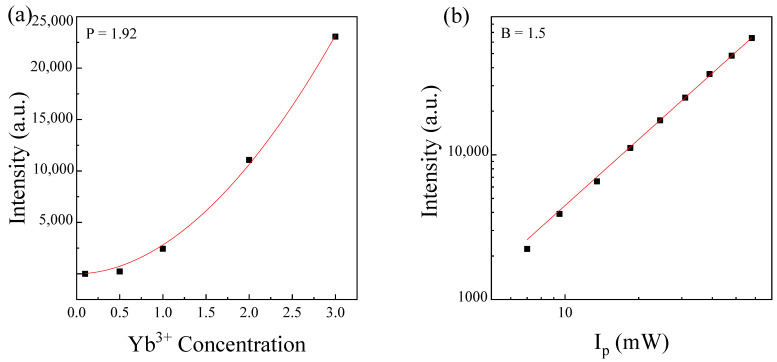
Tb^3+^ emission intensity as a function of Yb^3+^ concentration fitted to Equation (3) (solid line) (**a**). Plot of the ^5^D_4_→^7^F_5_ emission intensity as a function of pumping power (I_p_) at 980 nm obtained in a 2.5Tb^3+^-3Yb^3+^ co-doped phosphate glass (**b**).

In order to analyze the up-conversion mechanism, Figure 7a showed the luminescence intensity of the Tb^3+^ ions as a function of Yb^3+^ concentration. As can be seen, this luminescence increased with the Yb^3+^ concentration. Therefore, the energy transfer efficiency from Yb^3+^ ions to Tb^3+^ions increased with the increasing of Yb^3+^ ion content. If this up-conversion process is produced by a cooperative mechanism, then the relation between the up-conversion emission intensity, A_2_, and the donor concentration is determined by the following condition:

(6) $$A_{2}\propto C_{D}^{P}$$

where P is the power of the curve with a value of 2, according to Equation (3). Experimentally it is obtained as a value of P = 1.92, which is close to the expected value. It confirmed that the intensity of up-conversion emission was proportional to the quadratic donor concentration, as predicted in Equation (3) (indicating a two photon up-conversion mechanism produced by cooperative transfer).

In order to get deeper insight into the energy transfer mechanism involved in the up-conversion process, Figure 7b exhibited the dependence of the up-conversion luminescence intensity on near infrared excitation power for 2.5Tb^3+^-3Yb^3+^ co-doped phosphate glass. It is noteworthy that according to Equation (3), the relation between the up-conversion emission intensity, A_2_, and the pump power intensity can be expressed by the following condition:

(7) $$A_{2}\propto \Phi^{B}$$

Therefore, the slope obtained by fitting the experimental data was B = 1.5, which was close to the expected value of 2, confirming the expected quadratic dependence on the pump intensity mentioned in Equation (3) that indicated that a two photon up-conversion mechanism was involved [17].

#### 4.2.2. Temporal Evolution of the Up-Conversion Emission

**Figure 8:**
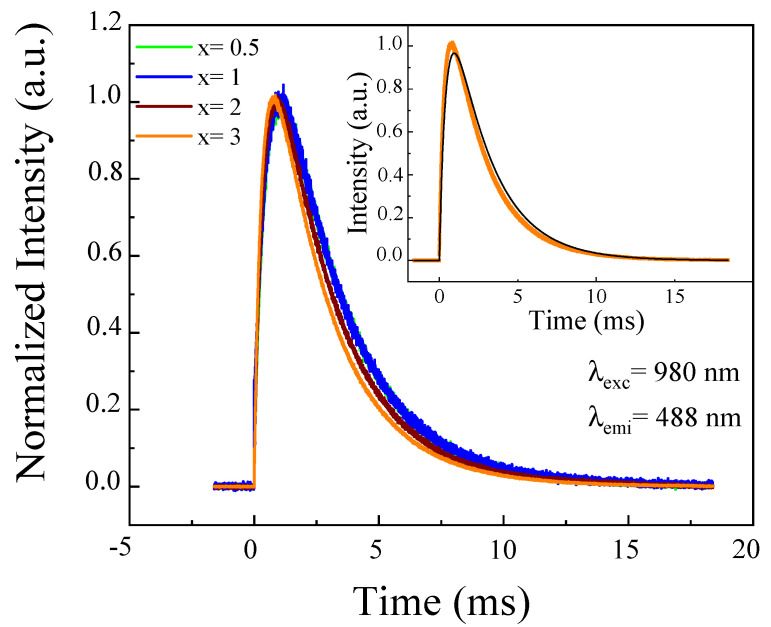
Temporal evolution of Tb^3+^ emissions at 488 nm obtained under excitation at 980 nm in 2.5Tb^3+^-xYb^3+^ co-doped phosphate glasses. The inset shows the fit of the decay curve for 2.5Tb^3+^-3Yb^3+^ co-doped phosphate glass to Equation (4).

The temporal evolution of the up-conversion emission was measured under direct excitation at 980 nm and detected at 488 nm (Figure 8). It can be seen that the temporal evolution of the Tb^3+^ up-conversion emission after excitation of Yb^3+^ ions showed a rise and decay of time. It was noticed that with the increasing of Yb^3+^ ions content, the rise time was fast, due to the short lifetime of the Yb^3+^ ions. The experimental results can be fitted to Equation (4), as exampled in the inset showing the fitting to the 2.5Tb^3+^-3Yb^3+^ sample. From this fitting, the pre-exponential factor $\frac{{\mathrm{WC}}_{A}Y_{2}{\left(0\right)}^{2}}{\frac{1}{\tau_{A}}-\frac{2}{\tau_{D}}}$ was estimated to be −1.7. This negative value was due to the donor lifetime τ_D_ = 0.9 ms being lower than the acceptor lifetime (τ_A_ *=* 2.6 ms). These values were consistent with the previous results for this sample of the Yb^3+^ and Tb^3+^ lifetimes of 0.9 ms and 2.6 ms, respectively. This result confirmed the cooperative energy transfer (CET) from a pair of Yb^3+^ ions to a Tb^3+^ ion.

In summary, the proposed scheme of the energy transfer mechanism (Yb^3+^→Tb^3+^) is presented in Figure 1. The ^5^D_4_ level could be populated through the CET from Yb^3+^ according to the following equation:

2 × Yb^3+^ (^2^F_5/2_) + Tb^3+^ (^7^F_6_)→2 × Yb^3+^ (^2^F_7/2_) + Tb^3+^ (^5^D_4_) (8)

Moreover, the ^5^D_4_ state can be excited to ^5^D_1_ level via an energy transfer from a third excited Yb^3+^ ion located in close proximity to a pair of interacting Yb^3+^ ions [17]. Then, the ^5^D_1_ rapidly relaxes to the ^5^D_3_, from which the weak emission bands are obtained, shown in Figure 6.

## 5. Conclusions

A series of 2.5Tb^3+^-xYb^3+^ co-doped phosphate glasses were prepared in order to obtain energy conversion via down- and up-conversion processes between visible and near infrared radiation. The emission spectra under excitation at 488 nm showed an increasing of Yb^3+^ emission intensity that confirmed the existence of energy transfer from Tb^3+^ to Yb^3+^. However, when Yb^3+^ concentration exceeded 1 mol%, the near infrared emission intensity began to reduce. The concentration quenching of Yb^3+^ became a serious problem to limit the energy transfer, which can be explained by the energy migration between Yb^3+^ ions. This energy can be transferred to a trap or to lower levels of Tb^3+^ ions. Alternatively, the up-conversion emission spectra were obtained under 980 nm excitation. It was observed that the emission intensity corresponding to the Tb^3+^ ions transitions ^5^D_4_→^7^F_J_ increased, as Yb^3+^ concentration increased, confirming a cooperative energy transfer from two excited Yb^3+^ ions to a Tb^3+^ ion. This result was supported by the influence of Yb^3+^ concentration on up-conversion emission intensity, as well as the dependence of up-conversion luminescence intensity on near infrared excitation power. The temporal evolution of the Tb^3+^ up-conversion emission showed a rise time τ_D_/2 and a decay time τ_A_, which was in good agreement with the theoretical model. Consequently, the level ^5^D_4_ was populated by cooperative energy transfer process from a pair of Yb^3+^ ions.

## Data Availability

The data presented in this study are available on request from the corresponding author.
